# Primary Genetic Investigation of a Hyperlipidemia Model: Molecular Characteristics and Variants of the Apolipoprotein E Gene in Mongolian Gerbil

**DOI:** 10.1155/2014/410480

**Published:** 2014-06-01

**Authors:** Yuehuan Liu, Jiusheng Wu, Qiaojuan Shi, Honggang Guo, Huazhong Ying, Ningying Xu

**Affiliations:** ^1^College of Animal Sciences, Zhejiang University, Hangzhou 310058, China; ^2^Zhejiang Academy of Medical Sciences, Hangzhou 310013, China

## Abstract

The objective of this work was to establish a novel Mongolian gerbil (*Meriones unguiculatus*) hyperlipidemia model and to investigate its susceptibility genetic basis. Two rodent (gerbil and rat) hyperlipidemia models were induced by feeding a high fat/high-cholesterol (HF/HC) diet. There were significant increases of serum total cholesterol, triglycerides, low-density lipoprotein cholesterol (LDL-C), and high-density lipoprotein cholesterol (HDL-C) in gerbils within a 4-week modeling period. About 10–30% of >8-month-old individuals developed hyperlipidemia spontaneously. The apolipoprotein E (ApoE) gene was cloned by merging a sequence of rapid amplification of cDNA ends (RACE) and nested polymerase chain reaction products. The results revealed an open reading frame of 948 bp, encoding a protein of 298 amino acids. The gene without a 5′-UTR region in the first intron was highly homologous to human Apo-A-I and rat Apo-A-IV. The distribution of expression of the ApoE gene in liver, brain, heart, lung, kidney, and adrenal gland was detected by an ABC immunohistochemical procedure. Three single nucleotide polymorphisms (SNPs; C97T, G781T, and A1774T) were first found using PCR-single-strand conformation polymorphism (PCR-SSCP) in a closed population containing 444 animals. Correlation analysis confirmed that new SNPs , age, and gender were associated significantly (*P* < 0.05) with hyperlipidemia.

## 1. Introduction

Current rodent models for hyperlipidemia include induced rats, guinea pigs, Syrian hamsters, and gene-knocked-out mice worldwide [1]. Hyperlipidemia is major risk factor for the development of cardiovascular disease. Despite the multifactorial pathogenesis of hyperlipidemia, high intakes of calories and fats (cholesterol-rich fats and saturated fatty acids) are widely considered as major contributing factors [2]. Although rats and mice have been extensively used in biomedical research to generate data on metabolic diseases, they have limitations as models of lipid metabolism in humans (for example, lipid metabolism particularly in changes of lipoproteins and serum cholesterol levels in response to dietary factors differ significantly between rats and humans) [3]. Thus, exploiting a new experimental animal model in studying hyperlipidemia promotes extrapolation of data since fatty acids and cholesterol play important roles in the etiology of diseases.

Early work with an induced hyperlipidemia model in rodents (rat, mouse, and gerbil) fed a high fat/high-cholesterol (HF/HC) diet showed the Mongolian gerbils (*Meriones unguiculatus*) model could be induced within 1 week without adding lard, egg yolk powder, or drugs to restrain thyroid activity [4, 5]. Feeding an HF/HC diet (included 2% (w/w) cholesterol and 7% (w/w) lard) can induce a prompt increase of total cholesterol and LDL-C concentration in peripheral blood and liver, which remains stable within 4 weeks [6]. Feeding an HF/HC diet also led to a significant increase in serum total cholesterol (TC), triglyceride (TG), low-density lipoprotein cholesterol (LDL-C), and high-density lipoprotein cholesterol (HDL-C) in gerbils, which were found in a time-dependent manner during 0~16 weeks' feeding. Hepatic lipid vacuolization and even fibrosis in gerbils were greatly formed in response to the high fat diet with the characteristic of serum LDL-C increase, while those index remained unchanged in rats. Furthermore, serum lecithin cholesterol acyl transferase (LCAT) activities in the hyperlipidemia gerbils were significantly higher than those in the normal ones, which were also in line with increased LDLC-TG secretion rate and impaired hepatic function in gerbils in response to the HF/HC diet. Therefore, gerbils were considered to be more sensitive to high fat diet and less time-consuming in forming hyperlipidemia, with similar response in increased LDL-C levels to cholesterol as human (there are two distinct features in lipid content in this model compared to human. One is its LDL-based particle. Another is over 30% free cholesterol in serum) [7]. Moreover, there are about 10–30% Z:ZCLA gerbils (a new experimental strain in China) of over-8-month-old gerbils (named old gerbils) prevalent for hyperlipidemia (hypercholesterolemia). But their genetic basis is unknown.

The human apolipoprotein E (ApoE) gene is located on 19q13.2, where it is linked with ApoC I, II, and IV. The 3.7 kb gene, which includes 4 exons and 3 introns, encodes a 34,200 Da glycoprotein composed of 299 amino acids [8]. As a major protein involved in lipoprotein metabolism and cardiovascular disease, ApoE might bidirectionally adjust action under the interaction of diet as environmental factors and genetic susceptibility, which has been detected in a variety of tissues, including liver, kidney, and brain [9]. It is well documented that the polymorphism and sequence variation of the ApoE gene have a strong correlation with lipid metabolism [10], cardiovascular diseases, cerebrovascular diseases, Alzheimer's disease, brain trauma, immune regulation, nerve regeneration and repair, and other forms of physiological activity [11, 12]. Up to now, the role in many aspects on ApoE has come from human cell models [13] and ApoE gene-deficient mice [14]. The hyperlipidemia and atherosclerosis animal pathogenesis and how ApoE affects hyperlipidemia and atherosclerosis are not entirely clear.

There has been no report of the relationship between the ApoE gene and cholesterol metabolism in Mongolian gerbils. In order to identify the genetic basis (discovered major genes or functional SNPs) and sequence structure and their effects on cholesterol metabolism, a total of 534 gerbils (model group and breeding population) were used to investigate the characteristics of the entire ApoE gene sequence, the polymorphism, and its relationship with the hyperlipidemia phenotype.

## 2. Materials and Methods

### 2.1. Animals and Diets

Sixty male gerbils (*Meriones unguiculatus*) of 50–70 g body weight, 60 male Sprague Dawley rats of 180–200 g body weight, and 30 > 8 months old gerbils, referred to here as old gerbils (manifested hyperlipidemia spontaneously), of 110–150 g body weight were obtained from the Zhejiang Center of Laboratory Animal, China. Five animals were housed together in a standard cage and kept at a temperature of 20–26°C with a 12 h light/12 h dark photoperiod. All experiments were done in accord with local guidelines and were approved by the Ethics Committee for Research on Laboratory Animal Use of Zhejiang Academy of Medicine Sciences. All animals (rats and gerbils) were given access* ad lib* to a commercially available standard diet (Zhejiang Center of Laboratory Animals, China) for 1 week before the experiment was started. The standard diet was produced in accordance with GB14924-2010 standard. It is composed of water and other volatile substances *⩽* 10%, crude protein *⩾* 18%, *⩾* 4% crude fat, crude fiber *⩽* 5%, crude ash *⩽* 8%, calcium 10–18 g, total phosphorus 6–12 g, calcium and phosphorus ratio 1.2–1.7, gerbils nutrition standards 4th edition (National Academy Press, Washington, D.C. 1995; 2–5% crude fat, crude protein 16–25%, calcium 5.0 g, and phosphorus 3.0 g). From day zero of the experiment, control animals (*n* = 30, ZC group) and the old gerbils group (*n* = 30, LN group) were fed the standard diet, and all other rats and gerbils (*n* = 30, GZ group) were fed an HF/HC diet composed of 80.5% (w/w) standard diet, 2% (w/w) cholesterol, 7% (w/w) lard, 10% (w/w) yolk powder, and 0.5% (w/w) bile salts [7].

### 2.2. Modeling, Sampling, Biochemical Testing, and Statistical Analysis

The influence of the different diets was observed during 4 weeks. During this time, we recorded body weight. After withholding food for 12 h, all animals were sacrificed by anesthesia with carbon dioxide; whole blood samples were collected for biochemical analysis and two small pieces of each of liver, brain, heart, spleen, lung, kidney, and adrenal gland were collected. One piece of each tissue was kept at −80°C for gene cloning and the other piece was used for routine histology tests (staining with Oil Red O and H&E) and ApoE immunohistochemical analysis. Serum was extracted for the detection of triglycerides (TG), total cholesterol (TC or CHO), high-density lipoprotein cholesterol (HDL-C), and low-density lipoprotein cholesterol (LDL-C).

### 2.3. To Acquire the Whole Sequence of the ApoE Gene Encoding Area

DNA was extracted with a tissue genomic DNA extraction kit (Shanghai Invitrogen, China). Total RNA were extracted with TRIzol reagent (Shanghai Invitrogen, China) and quantified by spectrophotometry. First strand cDNA was synthesized using a reverse transcriptase kit (Promega) and the middle region of the gene was cloned with primers APO-F1/APO-R1 and APO-F2/APO-R2. Rapid amplification of cDNA ends (RACE) was used to obtain the 5′ and 3′-flanking regions with the FirstChoice RLM-RACE Kit (Ambion). An additional four pairs of nested primers (APO-intron-F1—APO-intron-R8) were designed to amplify the introns. All amplified products were sequenced and constructed to one contig. A primer pair termed ApoE-full was designed to validate the contig and used to screen the SNP in the gerbil experimental population; 5 overlapping primer pairs were designed for PCR-SSCP (PCR-single-strand conformation polymorphism). All primers are given in Table 1. The PCR protocol was as follows: all reactions were done in 25 *μ*L volumes containing 0.25 *μ*L of LATaq DNA polymerase. The reaction sequence was 94°C for 4 min, then 35 cycles at 94°C for 30 s, 55–58°C for 30 s, 72°C for 45 s, followed by 72°C for 10 min and then 10°C insulation (Mastercycler Pros, Eppendorf).

Nine reference sequences from GenBank (human, M10065 and af261279; mouse, d00466; rat, J02582; chimpanzee, af200499; cattle, DQ538523; gorilla, AF200502; pig, u70240; and gibbon, AF200508) were used to calculate the phylogenetic tree with DNAstar software. Sequences of human (NC_000019), mouse (NC_000073), rat (J02582), tree shrew (AF303830), and rabbit (NM_001082643) from GenBank were used to compare the mRNA structure. The structure was compared with a RNA structure software (version 5.1).

### 2.4. Immunohistochemical Analysis

Tissue samples collected from gerbil groups ZC, GZ, and LN were used for immunohistochemical analysis of ApoE using the SABC kit (Booster Bioengineering Institute, Wuhan, China). Positive cells showed yellow or brown particles or clumps in the cytoplasm with a blue-stained nucleus, photographed with a 200X magnification objective lens.

### 2.5. Polymorphism Detection, Allele Frequency, and Association Analysis

DNA samples from the gerbil ZC, GZ, and LN groups were used to verify the gene contig and screen SNP. Polymerase chain reaction-single-strand conformation polymorphism (PCR-SSCP) was used to analyze DNA samples from a total of 444 animals; these animals were further distributed into several subgroups according to generation (45, 46, 47, 49), age period (149 <8 months old, 82 9–17 months old, 46 18–26 months old), and gender (♀, ♂). The PCR products were separated by PAGE, stained with silver, and photographed with a gel imaging system (Bio-Rad).

### 2.6. Correlation Analysis of ApoE Gene SNP with Hyperlipidemia of Gerbils

All data were analyzed by GLM procedure of SPSS16.0 software and presented as Mean ± SD. Correlation analysis was used to analyze the difference between SNP genotype distributions among groups. Tukey's multiple comparison was conducted to detect the difference among means. Biochemical indexes were compared by the 2-tailed Student's* t*-test. The statistically significant difference was set at *P* < 0.05. Genotypes and allele frequencies of three SNPs were computed by EXCEL software, and a Chi-square test was used to detect the significant difference.

The model used to analyze the correlation between the individual's genotype and hyperlipidemia trait was as follows:

(1) $$\begin{array}{c} Y_{ijklm}=\mu +S_{i}+F_{j}+G_{k}+l_{l}+Pm+\beta *X_{ijklm}+e_{ijklm}, \end{array}$$

where *Y*_*ijklm*_ is trait phenotypic value, *μ* is group average, *S*_*i*_ is sex effect, *E*_*j*_ is environment effect, *G*_*k*_ is genotype effect, *l*_*l*_ is generation effect, *P*_*m*_ is age effect, *β* is regression coefficient for hyperlipidemia trait, *X*_*ijklm*_ is hyperlipidemia indicator as covariate, and *e*_*ijklm*_ is residuals value.

## 3. Results

### 3.1. Biochemical Index and Pathology of Gerbil Hyperlipidemia Model

The serum level of total cholesterol (TC or CHO), triglycerides (TG), LDL-C, and HDL-C is given in Table 2. The serum TC level of the gerbil GZ group multiplied rapidly to 10-fold higher compared to the gerbil ZC group and sustained a 5-fold increase over 4 weeks (*P* < 0.01) but the increase was only 2-fold for the gerbil LN group. LDL-C and HDL-C of the gerbil GZ group increased rapidly to 4-fold greater compared to the gerbil ZC group and remained stable over four weeks. By contrast, the gerbil ZC group and rats were basically unchanged. The increased level of cholesterol was due mainly to elevated LDL-C rather than HDL-C. The HDL-C/TC ratio of the gerbil GZ group was lower compared to the gerbil ZC and rat groups. It is easy to conclude that the serum cholesterol transport in the gerbil GZ group depended largely on LDL-C, which is analogous to human.

**Figure 1:**
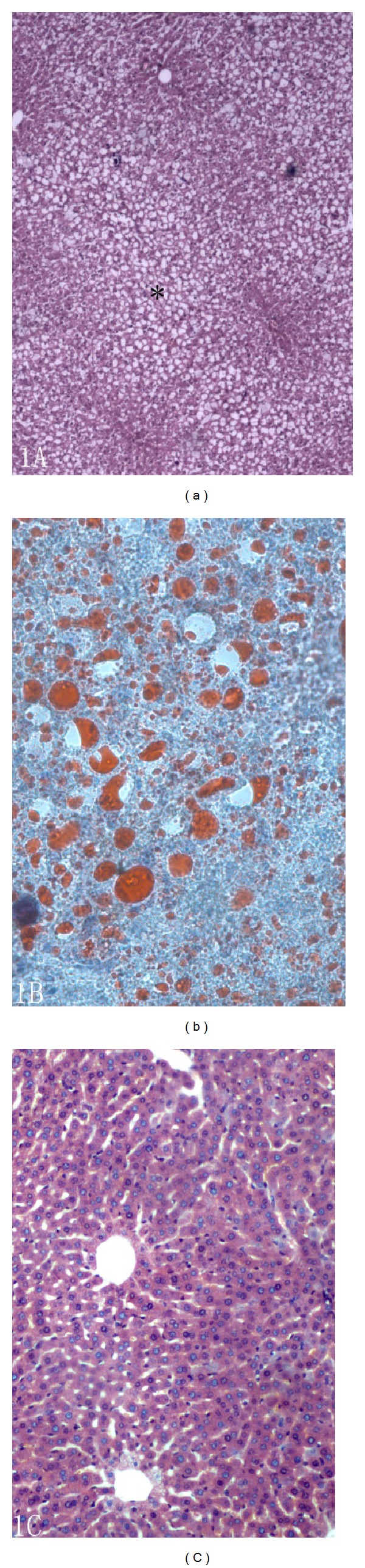
(a) Pathological section (HE staining) from Mongolian gerbil hyperlipidemia (Olympus, 10X objective lens); (b) pathological section (oil red staining) from Mongolian gerbil hyperlipidemia (Olympus, 40X objective lens); (c) pathological section (HE staining) from controlled group Mongolian gerbil (Olympus, 40X objective lens).

The GZ group gerbils were heavier compared to the ZC group. Body fat deposition, fatty liver, and swollen spleen were observed in GZ group gerbils. Staining with Oil Red O and H&E suggested that after 4 weeks, gerbil liver tissue exhibited significant fatty degeneration of liver cells, which was seen also in LN group gerbils (Figure 1). Liver lobules of ZC group gerbils and rats were evident; no lipid deposition was seen by staining hepatic cells.

### 3.2. Molecular Cloning and Sequence Analysis of the ApoE Gene in the Mongolian Gerbil

**Figure 2:**
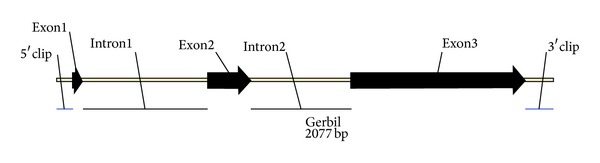
The genomic structure and sequences of ORF of the gerbil ApoE gene. The box structure, the gene sequence, includes the 5′-UTR (65 bp), three exons (44 bp, 181 bp, and 726 bp, resp.), two introns (521 bp and 421 bp, resp.), and 3′-UTR (119 bp).

The cDNA sequence (CDS) of the gerbil ApoE gene is 1135 bp long. The full-length gene is composed of a 5′-UTR (65 bp), a 3′-UTR (119 bp), 3 exons (44 bp, 181 bp, and 726 bp), and 2 introns (521 bp and 421 bp), which were constructed to a 2077 bp contig (accession number EU834053).  Figure 2 shows the structure of the complete gene. The typical start codon (ATG) and a stop codon (TGA) were located on the ApoE gene sequence but the exon/intron boundaries were in conflict with the GT/AG rule. The poly-(A) signal site (AATAAA) was located 30 bp upstream from the poly-(A) tail. Four-copy ATTTA motifs, which are known as the most important determinants of RNA stability, were located at 2 intron sequences. The gerbil ApoE gene sequences were registered in GenBank (accession number EU834053). Clustering analysis indicated gerbil, mouse, and rat are combined into a separate group. The homology of ApoE CDS of gerbils with mouse and rat reached 86%, 79.2% with human and < 78% with nonhuman primates or domestic animals. The Mongolian gerbil ApoE mRNA open reading frame (ORF) was 951 bp, the free energy was −31.0 kcal, the stem bases were 552 bp, and the ring bases were 501 bp; the human ORF was 954 bp, the free energy was −625.80 kcal, the stem bases were 536 bp, and the ring bases were 610 bp.

A gene that coded a protein of 316 amino acids was predicted, which included an 18-amino-acid signal peptide. The sequence contains 50 negatively charged residues (Asp + Glu) and 47 positively charged residues, which form 3 *α*-helical regions and a signal peptide. The LDL-R binding site (LRKMRKRLLR) was found in positions 137–146 of the mature protein. The detailed sequence of the mature protein was registered in GenBank (accession number EU834053).

### 3.3. Distribution of ApoE Protein in the Mongolian Gerbil 

**Figure 3:**
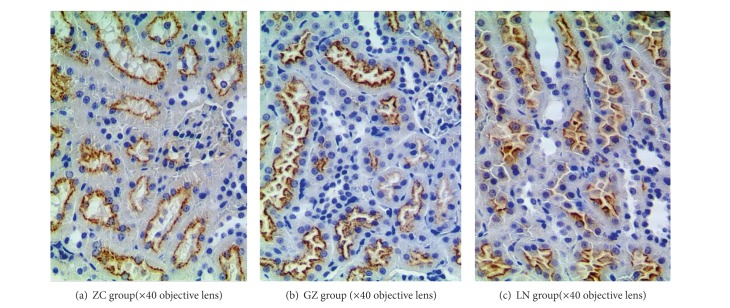
Presented the immunostaining results of kidney in each group. The positive products were distributed in the surface of the proximal convoluted tubules in the GZ, LN and ZC group.

Immunohistochemical staining showed ApoE-positive reactions in the liver, brain, heart, lung, kidney, and adrenal gland of the gerbil. In the brain, ApoE-positive products were distributed in both white and gray matter and staining was located mainly on the astrocytes. In the liver, all hepatocytes contained weakly immunoreactive granules. In the lung, ApoE-positive products were stained mainly in some pneumocytes lining the alveolar septa, especially in some type II pneumocytes. In the kidney, ApoE was located mainly on the epithelium in most of the proximal convoluted tubules. In the adrenal cortex, cells containing abundant granular reaction products were distributed mainly in the zona fascicularis and zona reticularis. In the zona glomerulosa, only the plasma membrane was stained. Expression of ApoE was observed in brain and kidney of the gerbil GZ and LN groups but the reaction was feeble and sparse in the ZC group. These results suggested that a variety of tissues and cells in the gerbil can synthesize and secrete ApoE (Figure 3).

### 3.4. SNP Screening and Analysis of ApoE Gene Polymorphism in the Mongolian Gerbil

**Figure 4:**
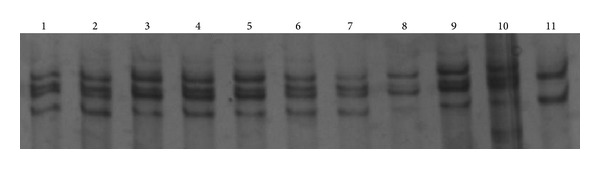
Genotypes of G781T SNP analyzed by PCR-SSCP, genotype: 1,9GT; 8TT; 11GG.

Sequencing results of SNP screening and PCR-SSCP indicated that base mutations were detected by primer pairs 1, 2, and 5. The polymorphic sites were located at positions 97 (C → T), 781 (G → T) (Figure 4), and 1774 (A → T), which resulted in three changes of the amino acid sequence: at site 11 (signal peptide) Ala was mutated to Val, at site 65 leu was replaced to Leu, and at site 256 Lys was mutated to Met. Primer pair 1 distinguished 3 genotypes (CT, CC, and TT), primer pair 2 detected 3 genotypes (GG, GT, and TT), and primer pair 5 detected 2 genotypes (AT and AA) in the test group. The genotype and gene frequencies are given in Table 3. At site 97, the frequency of allele C reached > 0.95 and was the dominant position among generations. At sites 781 (G → T) and 1774 (A → T), mutation genotype was relatively equilibratory, owing mainly to the possession of a considerable number of heterozygotes; the 781 heterozygotes accounted for the majority.

### 3.5. Association Analysis of the ApoE Gene by PCR-SSCP Polymorphism with Hyperlipidemia

The mutation effects in three sites were analyzed as follows (Table 4). In the SNPC97T site, 3 genotypes were correlated with body weight, CHO, and TG (*P* < 0.01). The SNPG782T was correlated with body weight, CHO, HDL, LDL, and TG (*P* < 0.01). The SNPA1774T site led to a TG difference. The ApoE gene was associated significantly with age (*P* < 0.01). Additionally, gender difference can display a change in serum levels of lipids (*P* < 0.05). In males, body weight, CHO, HDL, and TG were significantly higher (*P* < 0.05) compared to females.

## 4. Discussion 

Atherosclerosis or fatty liver disease is caused mainly by hyperlipidemia in animal models. An HF/HC diet is often used to induce a fatty liver disease model in Sprague Dawley or Wistar rats for evaluation of drug and lipid metabolism [15, 16]. However, these rat models have some limitations in pathological practice. Recently, several expensive genetic animal models (such as ApoE gene knock-out mice or LDL-R gene-deficient mice) were used for human hyperlipidemia and atherosclerosis research. In the gerbil model, cholesterol was added to the HF/HC diet to a final concentration of 2% (w/w) (about 10-fold greater compared to endogenous amounts) [1]. The plasma content of total cholesterol (CHO) and LDL-C increased to > 500 mg/dL and > 300 mg/dL, respectively, which is similar to the plasma level detected in LDL-R gene-deficient mice [17]. A short modeling period and its similarity to human hyperlipidemia led to the gerbil being the preferred model in pharmacology experiments [7, 18]. Hyperlipidemia might occur spontaneously in the gerbil under certain nutritional conditions (refer to GB14924-2010 Chinese National Standards), which include many types of cholesterol and different fatty acids. It is likely that the expression of ApoE gene constantly regulated the uptake rate of LDL-C and sterol by the liver, the degradation of the rate-limiting enzymes for cholesterol, biosynthesis of the LDL-C receptor and the volume of cholesterol esters. When the levels of exogenous and endogenous cholesterol are decreased, it is necessary to increase circulating levels of LDL-cholesterol to maintain steroidal homeostasis [19]. There are differences between the disorders of lipid metabolism in the rat and mouse models induced by the HF/HC diet. Two different clearance pathways (LDL-R/ApoB-100 and chylomicrons particulate/ApoB-48) can modify the ability to deal with the level of dietary cholesterol intake [20, 21]. Because the gerbil has perfect ability in cholesterol intake [22], it is likely that there exists an LDL-R and ApoB-100 clearance pathway. It is unclear whether ApoB-48 and/or chylomicrons particulate pathways exist in the gerbil.

The gerbil ApoE gene contains 3 exons and 2 introns encoding a protein of 298 amino acids. The ApoE gene has 4 exons separated by 3 introns encoding a protein containing a signal peptide in human, pig, rat, mouse, and dog [23–27]. The gorilla ApoE gene has 5 exons and 4 introns [28]. Quantitative sequence comparisons show that Apo-A-I, -A-IV, and E have evolved from a common ancestral gene [29]. The human Apo-A-I and Apo-A-IV genes in rat and mouse consist of 3 exons and 2 introns, and the first exon encodes a 5′-UTR and most of the signal peptide. Thus, the Apo-A gene has undergone multiple deletions that might affect its physiologic function, which is in contrast to all other known apolipoprotein genes [30, 31]. This work showed the gerbil ApoE gene is similar to Apo-A gene in rat or mouse.

Comparison of the mRNA primary structure of ApoE in several species shows the ORF of the gerbil gene is only 1 amino acid shorter compared to human. The first amino acid that combined with the LDLR, which is critical for gene transcription and expression, is glutamine in gerbil and human [32]. A single cysteine residue is found within the signal peptide region of the rodent but there is no cysteine residue in the mature coding region. Met was found instead of Leu in the gerbil, as in the rat, mouse, and tree shrew (*Tupaia glis*), which might affect the function of the LDL receptors [33]. The human ApoE protein has Arg (encoded by UGC) instead of Cys residues associated with type 3 hyperlipidemia [12]. Moreover, there are 10 CGC codes and 20 CGG codes in the gerbil ApoE, which is greater compared to rat, mouse, human, gorilla, and baboon [26, 28]. In addition, the deletion of a 5′-UTR region of the intron might influence the regulation of expression. The result suggested that the structure and function of ApoE in rodents are analogous to the E4 variant of human plasma ApoE [34].

ApoE can be synthesized and secreted in various mammalian tissues, including liver, brain, spleen, lung, ovary, kidney, and muscle [35]. Approximately 75% of ApoE in blood is synthesized by the liver, followed by the brain [36], and this study confirmed by immunohistochemistry that ApoE is upregulated/expressed in many organs and tissues in the gerbil and is highly expressed in the brain and kidney of gerbils with hyperlipidemia.

Mongolian gerbil is more susceptible to high fat diet and less time-consuming in forming hyperlipidemia during our many experiments or research works in many years, with similar response in increased LDL-C levels to cholesterol as human. Moreover, we found for the first time that some old individuals manifest a spontaneous hyperlipidemia in our close breeding population. It is crucially important to evaluate a new gerbil hyperlipidemia model, so that the highly expressed genes in diseased organs (such as liver) will be studied and major genes or functional SNPs will also be discovered. SNPs of the human ApoE gene show significant differences among nations or areas [37, 38]. There is no age or sex difference in the distribution of genotype frequencies but there are differences in genotype frequency among diverse ethnicities [39]. There are few reports of SNPs in rat and mouse and only 7 of SNPs in pig [24]. SNPs within the promoter region exert a marked influence on transcription of the porcine ApoE gene [40]. However, the ApoE gene is closely associated with porcine plasma ApoE protein, LDL and cholesterol concentration, atherosclerosis, and Alzheimer's disease. Three SNPs (97 (C > T), 781 (G > T), and 1774 (A > T)) mutations in the gerbil did not result in a change of the polarity of amino acids.

## 5. Conclusion

Mongolian gerbil might be a potential model for drug assessment and lipid metabolism. The apolipoprotein E (ApoE) gene includes an open reading frame of 948 bp, encoding a protein of 298 amino acids, which is analogous to human Apo E4. ApoE gene is upregulated/expressed in many organs and tissues in the gerbil and is highly expressed in the brain and kidney of gerbils with hyperlipidemia. Three new SNPs, age, and gender were significantly associated with hyperlipidemia.

## Figures and Tables

**Table 1:** Primers listed in this paper.

Primer name	Primer sequence	Size (bp) and location
APO-F1	5′ CCSTGCTGTTGGTCMCATT 3′	Degenerate primers (nest primer)
APO-R1	5′ GAGGCCTGTATCTTCTCC 3′	600 bp
APO-F2	5′ GAGCYGGAGGTGACAGAT 3′	
APO-R2	5′ GGCGMTGCATGTCTTCCACTA 3′	
APO-E-R1	5′ GCT CCT TTG TGT AAG CCT TCA C 3′	5′RACEprimer (nest primer)
APO-E-R2	5′ ATG GTG TCC TCT ATC AGA ACC GTC 3′	400 bp
APO-E-F1	5′ CAA GAT GGA GGA GCA GAC ACA 3′	3′RACEprimer (nest primer)
APO-E-F2	5′ GGA GGA GCA GAC ACA GCA GAT 3′	300 bp
APO-intron-F1	5′ ATGAAGGCTCTGTGGGCTG 3′	Fish for ApoE introns
APO-intron-R1	5′ AAGCCTTCACTTCTGTCATGGTGT 3′	(nest primer) 195 bp
APO-intron-F2	5′ GTCGCGTTGTTGGCAGGAT 3′	
APO-intron-R2	5′ GTCCTCTATCAGAACCGTCAG 3′	
APO-intron-F3	5′ TGCAGACGCTGTCTGACCA 3′	Fish for ApoE introns
APO-intron-R3	5′ CCAGCATGGTCTGTACCTC 3′	(nest primer) 169 bp
APO-intron-F4	5′ GACGGTTCTGATAGAGGACAC 3′	
APO-intron-R4	5′ GCCCAGTCTGTTGCGAAG 3′	
APO-intron-F5	5′ GACATGGAAGACCTTCGCAACA 3′	Fish for ApoE introns
APO-intron-R5	5′ CCTGGTTGCCCATCAGCT 3′	(nest primer) 287 bp
APO-intron-F6	5′ GCAGCGAGGTACAGACCA 3′	
APO-intron-R6	5′ GATGCGGGCACCCAAAGC 3′	
APO-intron-F7	5′ TGGTGGAGGAAGGTCGCC 3′	Fish for ApoE introns
APO-intron-R7	5′ GTAGGGAGGATGGGATTGGT 3′	(nest primer) 227 bp
APO-intron-F8	5′ GTCGGCTGGAGCTGATGG 3′	
APO-intron-R8	5′ GGGATTGGTAGCCACGGA 3′	
APO-Full-F1	5′ CCC GAA GGC TAA GGT TTT G 3′	Contig primer
APO-Full-R1	5′ ACC TGC TGG TCG TGG AT 3′	(Nest primer) 1800 bp
APO-Full-F2	5′ GGC TGG TTC ATC ACA GTT GTG 3′	
APO-Full-R2	5′ TGG AGA GGG ATC TTC ATT GAC TCT 3′	
liuF1	5′ GGTCGCGTTGTTGGCAGGTA 3′	SSCP primer, 410 bp
liuR1	5′ ATAGGACAGAGCTGAGACCA 3′	
liuF2	5′ AGACGCTGTCTGACCAGGTC 3′	SSCP primer, 449 bp
liuR2	5′ AGGGCCTAGACAGAAGGGCC 3′	
liuF3	5′ CTGGAGCTGATGGGCAACCA 3′	SSCP primer, 275 bp
liuR3	5′ GGGGTGGAGAGGGATCTTCA 3′	
liuF4	5′ CCCCCGAAGGCTAAGGTTTT 3′	SSCP primer, 225 bp
liuR4	5′ ATAAAAAAGCTGCTCGGGGC 3′	
liuF5	5′ CTGGAGCTGAT GGGCAACCA 3′	SSCP primer, 347 bp
liuR5	5′ AAGTAAGGGCCACCAGAGGG 3′	

*All primers were synthesized by Shanghai Sangon, China.

**Table 2:** The blood biochemical index of SD rat and Mongolian gerbil.

	GZ rat	ZC rat	GZ gerbil	ZC gerbil	LN gerbil
CHO (mg/dL)	255.29 ± 105.96**	103.66 ± 27.46	505.93 ± 266.51**	116.04 ± 33.65	230.92 ± 144.28*
TG (mg/dL)	89.64 ± 15.98	120.70 ± 55.91	87.86 ± 76.33	78.10 ± 31.95	1403.40 ± 959.9**
HDL (mg/dL)	38.25 ± 7.73	24.34 ± 6.96	751.55 ± 386.17**	72.64 ± 17.77	166.15 ± 55.77
LDL (mg/dL)	75.87 ± 22.06	81.68 ± 20.13	379.75 ± 201.94**	96.78 ± 17.03	96.78 ± 73.55

*Two-tailed Student's *t*-test was applied to calculate difference between treatments, **means *P* < 0.01, and *means *P* < 0.05.

**Table 3:** Genotypes and allele frequencies of three SNPs in ZCLA Mongolia gerbil population.

	Genotypes and allele frequencies of 3 SNPs												
	SNPC97T				SNPG781T					SNPA1774T			
	CC	CT	TT	C	GG	GT	TT	G	*χ* ^2^	AA	AT	A	*χ* ^2^
	*N*	*N*	*N*	%	*N*	*N*	*N*	%		*N*	*N*	%	
Generation													
45	46	0	2	0.9583	7	36	5	0.5208	10.19**	3	50	0.5283	38.74**
46	54	0	1	0.9818	2	49	0	0.5196	39.96**	0	33	0.5000	29.11**
47	73	0	3	0.9605	1	83	1	0.5000	73.42**	2	63	0.5154	53.76**
49	169	5	3	0.9689	4	197	2	0.5049	176.03*	13	192	0.5317	184.74**
Gender													
♀	200	5	3	0.9736	8	219	4	0.5087	182.16**	9	220	0.5197	191.99**
♂	142	0	6	0.9595	6	146	4	0.5064	115.18**	9	118	0.5354	115.18**
Age													
<8 months	214	5	4	0.9709	4	249	1	0.5059	230.70**	13	222	0.5277	184.74**
9–17 months	82	0	3	0.9647	3	80	2	0.5059	62.73**	2	66	0.5147	56.73**
18–26 months	46	0	2	0.9583	7	36	5	0.5208	10.19**	3	50	0.5283	38.74**

*χ ^2^ Test for Hardy-Weinberg equilibrium, *P* < 0.05, **P < 0.01.

**Table 4:** Association analysis between SNP genotypes and recorded traits

	Body weight	CHO	HDL	LDL	TG
	*X* ± SD	*X* ± SD	*X* ± SD	*X* ± SD	*X* ± SD
Gender					
♀	76.2 ± 21.9^B^	104.8 ± 69.2^b^	31.2 ± 23.5^b^	50.3 ± 42.6	648.5 ± 491.3^b^
♂	103.9 ± 25.0^A^	129.6 ± 78.5^a^	36.2 ± 34.6^a^	46.5 ± 31.0	758.3 ± 527.7^a^
Age					
<8 months	77.5 ± 23.8^B^	92.1 ± 35.6^c^	28.1 ± 6.9^B^	46.5 ± 38.7^B^	491.2 ± 263.8^B^
9–17 months	106.8 ± 22.3^A^	150.1 ± 98.2^b^	41.9 ± 49.6^A^	46.5 ± 31.0^B^	1046.8 ± 595.2^A^
18–26 months	104.8 ± 20.9^A^	173.3 ± 114.1^a^	46.2 ± 41.5^A^	65.8 ± 38.7^A^	1172.4 ± 680.4^A^
C97T					
CC	88.0 ± 26.7^B^	114.5 ± 73.5^B^	33.5 ± 30.8	46.5 ± 27.1	705.1 ± 490.7^b^
CT	62.7 ± 5.5^C^	75.0 ± 15.9^B^	24.2 ± 1.5	31.0 ± 7.7	245.5 ± 210.3^c^
TT	110.1 ± 32.3^A^	167.9 ± 104.0^A^	38.5 ± 16.9	61.9 ± 34.8	1001.1 ± 732.6^a^
G781T					
GG	113.3 ± 33.2^A^	200.4 ± 138.1^A^	54.2 ± 58.1^A^	73.5 ± 42.6^A^	1025.5 ± 714.8^A^
GT	86.4 ± 26.0^B^	109.5 ± 68.5^B^	31.9 ± 23.5^B^	46.5 ± 38.7^B^	669.1 ± 487.5^B^
TT	106.7 ± 22.7^A^	163.2 ± 106.4^A^	70.8 ± 108.8^A^	58.1 ± 34.8^A^	1136.0 ± 651.6^A^
A1774T					
AA	84.9 ± 22.7	106.4 ± 54.2	39.6 ± 49.2	46.5 ± 46.5	486.5 ± 359.4^a^
AT	85.6 ± 26.2	115.7 ± 78.9	33.5 ± 30.0	50.3 ± 42.6	701.0 ± 529.4^b^

*Estimated value is the least squares mean ± SD. Different lowercase letters a, b, and c indicate significant difference at 0.05 level; uppercase letters A, B, and C indicate significant difference at 0.01 level.
